# Differences in the prevalence of hospitalizations and utilization of emergency outpatient services for ambulatory care sensitive conditions between asylum-seeking children and children of the general population: a cross-sectional medical records study (2015)

**DOI:** 10.1186/s12913-017-2672-7

**Published:** 2017-11-15

**Authors:** Célina Lichtl, Thomas Lutz, Joachim Szecsenyi, Kayvan Bozorgmehr

**Affiliations:** 1 Department of General Practice and Health Services Research, University Hospital Heidelberg, Im Neuenheimer Feld 130.3, 69120 Heidelberg, Germany; 2 Center for Child and Adolescent Medicine, Department of General Pediatrics, Metabolism, Gastroenterology, Nephrology, University Hospital Heidelberg, Im Neuenheimer Feld 430, 69120 Heidelberg, Germany

**Keywords:** Ambulatory care sensitive conditions, Ambulatory care sensitive hospitalizations, ACS, ACSC, ACSH, Asylum seeker, Children, Minor, Pediatric, Preventable, Avoidable

## Abstract

**Background:**

Hospitalizations for ambulatory care sensitive (ACS) conditions are established indicators for the availability and quality of ambulatory care. We aimed to assess the differences between asylum-seeking children and children of the general population in a German city with respect to (i) the prevalence of ACS hospitalizations, and (ii) the utilization of emergency outpatient services for ACS conditions.

**Methods:**

Using anonymous account data, all children admitted to the University Hospital Heidelberg in 2015 were included in our study. A unique cost unit distinguished asylum seekers residing in a nearby reception center (exposed) from the children of the general population. We adapted international lists of ACS conditions and calculated the prevalence of ACS hospitalizations and the utilization of emergency outpatient services for ACS conditions, attributable fractions among the exposed (Afe) and the population attributable fraction among total admissions (PAF) for each outcome. Differences in the prevalence of each outcome between exposed and controls were analyzed in logistic regression models adjusted for sex, age group and quarterly admission.

**Results:**

Of the 32,015 admissions in 2015, 19.9% (6287) were from inpatient and 80.1% (25,638) from outpatient care. In inpatient care, 9.8% (622) of all admissions were hospitalizations for ACS conditions. The Afe of ACS hospitalizations was 46.57%, the PAF was 1.12%. Emergency service use for ACS conditions could be identified in 8.3% (3088) of all admissions (Afe: 79.57%, PAF: 5.08%). The odds ratio (OR) of asylum-seeking children being hospitalized for ACS conditions in comparison to the control group was 1.81 [95% confidence interval, CI: 1.02; 3.2]. The OR of the asylumseeking population compared to the general population for the utilization of emergency service use for ACS conditions was 4.93 [95% CI: 4.11; 5.91].

**Conclusions:**

Asylum-seeking children had significantly higher odds of ACS hospitalization and of utilization of emergency outpatient services for ACS conditions. Using the concept of ACS conditions allowed measuring the strength of primary care provided to this local asylum-seeking population. This approach could help to compare the strength of primary care provision in different locations, and allow an objective.

## Background

Access to health care for migrants seeking international protection in Germany is regulated by the Asylum Seekers’ Benefits Act (*"Asylbewerberleistungsgesetz"*). This law grants asylum seekers basic health care services. Benefits allocated for medical care cover necessary medical or dental treatment of acute illness and pain, including the provision of medication and bandages and necessary measures for convalescence, recovery or alleviation of disease or necessary services addressing consequences of illnesses [1]. Vaccination and “necessary preventive medical check-ups” are also to be provided. In addition, pregnant women and women who have recently given birth are entitled to “medical and nursing help and support” including midwife assistance. The respective federal state’s administrative regional council (*"Regierungspräsidium"*) covers the costs for health care for asylum seekers residing in reception centers. Asylum seekers undergo a mandatory health examination [2] focused on infectious diseases in reception centers, and reside there for up to 6 months until they are transferred to cities or communities based on specific dispersal policies. After transfer of asylum seekers to collective accommodation centers or decentralized accommodation, the social welfare office of the responsible district bears the incurred costs.

Health care in German reception centers is often provided on an irregular basis [3]. Due to reception centers often being located in remote geographical areas with insufficient local transport to health care providers, the availability of accessible health care services is also limited. In addition, asylum seekers may lack sufficient knowledge on their entitlements to health care, the underlying regulations and the structure of medical care (primary, secondary and tertiary care) due to lack of communication, language barriers or other reasons and therefore not make use of said entitlements [4]. As a result, these barriers can lead to delayed care, which results in costly treatment [5]. In the European Union (EU) / European Economic Area (EEA), Germany stands out as one of the countries with the most restrictive health care policy for migrant children [6]. In the last decade, several models of health care provision to asylum seekers in reception centers have emerged in Germany, aiming at overcoming the aforementioned barriers [7]. There is, however, a lack of approaches to objectively compare different models with respect to their performance. Comparisons are also challenged by a relative lack of individual-level data on health and health care utilization among asylum seekers. This information is not routinely collected in Germany across regions and health care sectors [8, 9], turning the effects of barriers on access to health care invisible.

On an international level ambulatory care sensitive (ACS) hospitalizations are increasingly used as an indicator for the availability and quality of ambulatory care [10]. The concept of ACS hospitalizations is based on the assumption that a deficit of timely and effective outpatient care can lead to avoidable hospital admissions and potentially preventable hospitalizations. ACS conditions have been defined [11] as those conditions for which “the provision of timely and effective outpatient care can help to reduce the risks of hospitalization by either preventing the onset of an illness or condition, controlling an acute episodic illness or condition, or managing a chronic disease or condition” [1].

As a proof of concept, we aimed to assess the differences between asylum-seeking children and children of the general population in a German city with respect to (i) the prevalence of ACS hospitalizations, and (ii) the utilization of emergency outpatient services for ACS conditions.

## Methods

### Context and setting of the study

In December 2014, a reception center for refugees was set up in Heidelberg-Kirchheim in the former United States Army installation ‘Patrick Henry Village’ with around 900 refugees [12]. The reception center was at first planned as a temporary processing point for asylum seekers in times of high immigration (*"Bedarfsorienterte Erstaufnahmestelle"* ). Consequently, the number of medical professionals available was low and medical care was provided by a commercial health services agency and consisted of around four-hour visits from a general practitioner for a continuously increasing number of residents. The length of stay of asylum seekers in reception centers at this point was legally up to 6 months, and on average the duration of stay was estimated at 3 months or longer (personal communication with state authorities). In July 2015, the number of residents had risen to nearly 2600 [13] and more than 6500 in August. In September 2015 the temporary reception centre was converted into the first ‘initial registration centre’ (“*Zentrale Erstregistierungsstelle”*) for the state of Baden Württemberg. This is where the initial registration of asylum seekers is administered, the medical inspection and X-ray (tuberculosis screening) are performed and an asylum application is submitted – and in some cases directly decided upon. From this centre, depending on their asylum decision or prognosis of it, the asylum seekers then move to decentralized accommodation or other processing points in the federal state. The length of stay in the reception centre was subsequently reduced due to increased in-migration and faster transfers to other processing points. In February 2016, the average stay of asylum seekers in the reception centre was 4 weeks or more (personal communication with state authorities). Since the establishment of this center, Heidelberg University Hospital has been increasingly involved in health care provision to asylum seekers, and in February 2016 a walk-in clinic was established on-site [7, 14].

### Design

We performed a cross-sectional study based on medical records of Heidelberg University Hospital in 2015. The records include all children, which were admitted to the hospital for inpatient- and outpatient care in the year 2015 (01/01/2015 to 31/12/2015).

### Participants

All children under the age of 18, which were admitted to the hospital in the year 2015, were taken into account. The children were classified into exposed (asylum-seeking children) and controls (children of the general population) based on the patients’ cost units. Children with the cost unit ‘regional council’ *("Regierungspräsidium")* were classified as asylum seekers. This cost unit uniquely identifies refugees who are accommodated in reception centres under federal state mandate. Children with the cost unit ‘statutory- or private insurance’ were classified as controls belonging to the general population. Children with other cost units (self-payers, social welfare office, etc.) were excluded from the study, since these cost units do not provide unique information on children’s residence status.

### Data collection and recruitment

Anonymous account data from all children treated in the year 2015 was acquired from the accounts department of Heidelberg University Hospital. The dataset contained information on year of birth, month of birth, sex, date of admission, date of discharge, primary and secondary diagnoses, Diagnosis Related Group case payments in the case of inpatient data, specialist organizational units, nursing organizational units and the cost unit of the patient.

### Measuring ambulatory care sensitive (ACS) hospitalizations

International lists of ACS conditions for children have previously been established in several studies, mostly on the basis of expert consensus procedures or analysis of discharge records. For this study we selected and compared seven previously conducted studies [15–21], which had identified and validated pediatric ACS conditions as such. If a condition was validated as an ACS condition in three or more of the seven studies, we included it in the final list. We added three further conditions (allergies & allergic reactions, gastritis and neonatal jaundice) based on the expertise of local pediatricians at Heidelberg University Hospital. The final ACS conditions list comprised 17 conditions with a total of 304 ICD-codes.

The selected conditions are: 1) Allergies & allergic reactions, 2) Asthma, 3) Convulsions, 4) Dental conditions, 5) Diabetes mellitus, 6) Failure to thrive, 7) Gastritis, 8) Gastroenteritis / dehydration, 9) Immunization-preventable diseases, 10) Inflammatory diseases of female pelvic organs, 11) Iron deficiency anemia / anemia, 12) Kidney- and urinary infections, 13) Nutritional deficiency, 14) Neonatal jaundice, 15) Severe ENT- infection 16) Skin infection, 17) Doctor’s orders have not been followed by patient.

All coded diagnoses for inpatient and outpatient-treatment were categorized according to the list of ACS conditions into a dichotomous variable indicating whether or not the hospitalization is considered as preventable (ACS hospitalization yes/no). The data were analyzed descriptively and analytically. In some cases the avoidability of diagnoses was considered debatable. In a sensitivity analysis, we excluded these diagnoses from the ACS conditions lists and repeated the analysis to assess whether our results were robust to this potential source of misclassification (data not shown).

### Data analysis

The descriptive analysis includes calculation of period means and standard deviations for interval-scaled variables and proportions for categorical and dichotomous variables stratified by residence status respectively. Differences in period means and proportions of underlying socio-demographic characteristics were analyzed by the t-test for independent samples and the chi-square test respectively (data not shown). For each outcome, we calculated attributable fractions among the exposed (*Afe)*, i.e. the refugee population (with hospitalizations for ACS), and the population attributable fraction (PAF) (calculated as proportion of cases exposed multiplied by the *Afe*). Differences in the prevalence of ACS hospitalizations and emergency use for ACS conditions between asylum-seeking children and children of the general population were examined by odds ratios (OR) and 95% confidence intervals (CI) obtained from single and multiple logistic regression analysis (adjusted for sex, age group and quarterly admission) using the statistic program STATA version 12.0.

## Results

### Descriptive results

Of the 32,015 admissions, i.e. hospital cases recorded in 2015, 6287 (19.9%) were from inpatient care and 25,638 (80.1%) admissions from outpatient care comprising a total of 21,742 children.

### Inpatient care

In inpatient care, there were a total of 90 admissions of asylum-seeking minors (1.4%) among the 6377 admissions. Of these, 55 (61.1%) patients were male and 35 (38.9%) were female, as opposed to 3390 (53.9%) males among the children of the general population and 2897 (46.1%) females (Table 1). The children were divided into six age groups, ranging from under 1 year of age to 1–3 years, 3–6 years, 6–10 years, 10–14 years and 14–18 years. In both population groups, the largest age group was that of children below 1 year of age with 33.3% among asylum-seeking children and 22.6% among children of the general population.

**Table 1:** Descriptive analysis of child admissions^a^: inpatient care (*N* = 6377 admissions) and outpatient care (*N* = 25,638 admissions)

		Inpatient care/Hospitalization						Outpatient care/Emergency service use					
		Residence status of children						Residence status of children					
		General population		Asylum seekers		Total		General population		Asylum seekers		Total	
		Freq.	(Col%)	Freq.	(Col%)	Freq.	(Col%)	Freq.	(Col%)	Freq.	(Col%)	Freq.	(Col%)
Sex	Male	3390	(53.9)	55	(61.1)	3445	(54)	13,375	(53.5)	403	(62.6)	13,778	(53.7)
	Female	2897	(46.1)	35	(38.9)	2932	(46)	11,619	(46.5)	241	(37.4)	11,860	(46.3)
	Total N(%)	6287	(100)	90	(100)	6377	(100)	24,994	(100)	644	(100)	25,638	(100)
Childrens’ age groups	14–18 years	1002	(15.9)	12	(13.3)	1014	(15.9)	3958	(15.8)	23	(3.6)	3981	(15.5)
	10–14 years	811	(12.9)	10	(11.1)	821	(12.9)	4068	(16.3)	54	(8.4)	4122	(16.1)
	6–10 years	911	(14.5)	5	(5.6)	916	(14.4)	4501	(18)	99	(15.4)	4600	(17.9)
	3–6 years	1055	(16.8)	9	(10)	1064	(16.7)	4972	(19.9)	149	(23.1)	5121	(20)
	1–3 years	1086	(17.3)	24	(26.7)	1110	(17.4)	4199	(16.8)	193	(30)	4392	(17.1)
	<1 year of age	1422	(22.6)	30	(33.3)	1452	(22.8)	3296	(13.2)	126	(19.6)	3422	(13.4)
	Total N(%)	6287	(100)	90	(100)	6377	(100)	24,994	(100)	644	(100)	25,638	(100)
Admissions per quarter of the year	1st quarter (Jan-Mar)	1688	(26.8)	16	(17.8)	1704	(26.7)	6826	(27.3)	106	(16.5)	6932	(27)
	2nd quarter (Apr-Jun)	1551	(24.7)	10	(11.1)	1561	(24.5)	6254	(25)	66	(10.3)	6320	(24.7)
	3rd quarter (Jul-Sep)	1525	(24.3)	26	(28.9)	1551	(24.3)	6156	(24.6)	236	(36.6)	6392	(24.9)
	4th quarter (Oct-Dec)	1523	(24.2)	38	(42.2)	1561	(24.5)	5758	(23)	236	(36.6)	5994	(23.4)
	Total N(%)	6287	(100)	90	(100)	6377	(100)	24,994	(100)	644	(100)	25,638	(100)
ACS condition	no	5680	(90.3)	75	(83.3)	5755	(90.2)	33,756	(92.1)	470	(70.5)	34,226	(91.7)
	yes	607	(9.7)	15	(16.7)	622	(9.8)	2891	(7.9)	197	(29.5)	3088	(8.3)
	Total N(%)	6287	(100)	90	(100)	6377	(100)	36,647	(100)	667	(100)	37,314	(100)
ACS conditions: Subcategories	Allergies & allergic reactions	17	(0.3)	0	(0)	17	(0.3)	76	(0.2)	1	(0.2)	77	(0.2)
	Asthma	13	(0.2)	0	(0)	13	(0.2)	64	(0.2)	2	(0.3)	66	(0.2)
	Convulsions	53	(0.8)	0	(0)	53	(0.8)	77	(0.2)	1	(0.2)	78	(0.2)
	Dental conditions	43	(0.7)	0	(0)	43	(0.7)	25	(0.1)	0	(0)	25	(0.1)
	Diabetes mellitus	35	(0.6)	1	(1.1)	36	(0.6)	721	(2)	1	(0.2)	722	(1.9)
	Failure to thrive	29	(0.5)	0	(0)	29	(0.5)	152	(0.4)	2	(0.3)	154	(0.4)
	Gastritis	48	(0.8)	0	(0)	48	(0.8)	126	(0.3)	8	(1.2)	134	(0.4)
	Gastroenteritis / Dehydration	28	(0.4)	5	(5.6)	33	(0.5)	1	(0)	0	(0)	1	(0)
	Immunization-preventable diseases	7	(0.1)	1	(1.1)	8	(0.1)	25	(0.1)	23	(3.5)	48	(0.1)
	Iron-deficiency anemia	17	(0.3)	0	(0)	17	(0.3)	18	(0)	1	(0.2)	19	(0.1)
	Kidney- and urinary infections	26	(0.4)	1	(1.1)	27	(0.4)	150	(0.4)	4	(0.6)	154	(0.4)
	Nutritional deficiency	3	(0)	0	(0)	3	(0)	23	(0.1)	0	(0)	23	(0.1)
	Neonatal jaundice	32	(0.5)	0	(0)	32	(0.5)	35	(0.1)	3	(0.5)	38	(0.1)
	Severe ENT-infection	218	(3.5)	7	(7.8)	225	(3.5)	1367	(3.7)	148	(22.2)	1515	(4.1)
	Skin infections	38	(0.6)	0	(0)	38	(0.6)	31	(0.1)	3	(0.5)	34	(0.1)
	Non-ACS conditions	5680	(90.3)	75	(83.3)	5755	(90.2)	33,756	(92.1)	470	(70.5)	34,226	(91.7)
	Total N(%)	6287	(100)	90	(100)	6377	(100)	36,647	(100)	667	(100)	37,314	(100)

^a^Differences in totals and column percent due to rounding

Overall, we found 622 admissions with hospitalization for ACS, i.e. in 9.8% of all admissions. Asylum-seeking children accounted for 15 (16.7% among population group) and children of the general population for 607 (9.7% among population group). The most common ACS condition of all 17 conditions among both population groups was the severe ENT infection with 2.2% overall, 7 admissions (3.3%) were recorded among asylum seekers and 225 (3.5%) among the control group.

The *Afe* of ACS hospitalizations was 46.57% [95% CI: 0.01; 0.7], the corresponding *PAF*, hereby referring to the whole population (total admissions with hospitalizations for ACS), was 1.12%.

### Outpatient care

Among the 25,638 admissions in outpatient care, there were a total of 644 admissions of asylum seekers (2.5%). 403 (62.6%) asylum-seeking patients were male and 241 (37.4%) were female (Table 1). The distribution between the sexes among the children of the general population was similar to that of inpatient care. The largest age group among asylum seekers was that of 1–3 year olds (30%) and that of 3–6 years olds (19.9%) among children of the general population.

Emergency service use for ACS conditions could be discovered in 3088 (8.3%) of admissions among both population groups. Asylum-seeking children accounted for 197 of these admissions, (29.5% among the population group) and children of the general population for 2891 (7.9% among population group). Severe ENT-Infections were yet again the most common overall and population-based ACS conditions among both populations (4.1% overall), 148 admissions (22.2%) were recorded among asylum seekers and 1367 (3.7%) among children of the general population.

The *Afe* of emergency service use for ACS conditions was 79.57% [95% CI: 0.76; 0.83], the *PAF* was 5.08%.

### Unadjusted single logistic regression estimates

The unadjusted simple regression estimates obtained from bivariate models showed statistically significant positive associations between asylum seekers’ residence status and both hospitalization for ACS conditions (Table 2) and utilization of emergency outpatient services for ACS conditions (Table 3).

**Table 2:** Crude and adjusted regression estimates for the association of exposure to residence status with hospitalization for ACS conditions in children, N = 6377 admissions

Explanatory variables OR, [95% CI]		Single regression models		Multiple regression models					
				Model I		Model II		Model III	
				Asylum seeker & sex		Asylum seeker, sex & age group		Asylum seeker, sex, age group & quarterly admission	
Residence status	General population	(Ref)		(Ref)		(Ref)		(Ref)	
	Asylum seekers	1.87	[1.05; 3.33]	1.87	[1.05; 3.34]	1.77	[1; 3.14]	1.81	[1.02; 3.2]
Sex	Male	(Ref)		(Ref)		(Ref)		(Ref)	
	Female	1.02	[0.85; 1.23]	1.03	[0.85; 1.24]	1.05	[0.85; 1.24]	1.05	[0.87; 3.2]
Age group	14–18 yrs	(Ref)				(Ref)		(Ref)	
	10–14 yrs	1.39	[0.94; 2.06]			1.40	[0.95; 2.06]	1.40	[0.95; 2.07]
	6–10 yrs	1.95	[1.27; 3]			1.97	[1.29; 3.02]	1.98	[1.29; 3.03]
	3–6 yrs	1.63	[1.14; 2.33]			1.65	[1.15; 2.35]	1.65	[1.16; 2.36]
	1–3 yrs	2.66	[1.91; 3.7]			2.65	[1.9; 3.69]	2.65	[1.9; 3.69]
	<1 yr	1.94	[1.4; 2.69]			1.94	[1.4; 2.68]	1.95	[1.41; 2.69]
Admission	1. Quarter	(Ref)						(Ref)	
	2. Quarter	1.00	[0.8; 1.26]					1.02	[0.81; 1.27]
	3. Quarter	0.84	[0.66; 1.08]					0.84	[0.66; 1.08]
	4. Quarter	0.98	[0.77; 1.24]					0.96	[0.75; 1.22]

*Ref* reference group, *CI* confidence interval

**Table 3:** Crude and adjusted regression estimates for the association of exposure to residence status with utilization of emergency outpatient services for ACS conditions in children, N = 25,638 admissions

Explanatory variables OR, [95% CI]		Single regression models		Multiple regression models					
				Model I		Model II		Model III	
				Asylum seeker & sex		Asylum seeker, sex & age group		Asylum seeker, sex, age group & quarterly admission	
Residence status	General population	(Ref)		(Ref)		(Ref)		(Ref)	
	Asylum seekers	4.89	[4.1; 5.85]	4.93	[4.12; 5.89]	4.77	[3.98; 5.72]	4.93	[4.11; 5.91]
Sex	Male	(Ref)		(Ref)		(Ref)		(Ref)	
	Female	1.06	[0.95; 1.17]	1.08	[0.97; 1.19]	1.07	[0.97; 1.19]	1.07	[0.97; 1.19]
Age group	14–18 yrs	(Ref)				(Ref)		(Ref)	
	10–14 yrs	0.78	[0.63; 0.96]			0.77	[0.62; 0.95]	0.77	[0.62; 0.95]
	6–10 yrs	0.65	[0.53; 0.8]			0.63	[0.52; 0.77]	0.63	[0.52; 0.77]
	3–6 yrs	0.88	[0.73; 1.05]			0.84	[0.7; 1.02]	0.85	[0.7; 1.02]
	1–3 yrs	1.19	[1; 1.42]			1.11	[0.93; 1.33]	1.11	[0.93;1.33]
	<1 yr	0.97	[0.8; 1.17]			0.91	[0.75; 1.1]	0.91	[0.75; 1.1]
Admission	1. Quarter	(Ref)						(Ref)	
	2. Quarter	0.79	[0.72; 0,87]					0.80	[0.73; 0.88]
	3. Quarter	0.78	[0.71; 0,86]					0.74	[0.67; 0.81]
	4. Quarter	0.87	[0.79; 0,96]					0.82	[0.75; 0.91]

*Ref* reference group, *CI* confidence interval

The odds of hospitalization for ACS conditions for minor asylum seekers in inpatient care were 1.87 times [95% CI: 1.05; 3.33] the odds of children of the general population (Table 2). Children between the age of 1 and 3 years had the highest chance of admission for ACS conditions with an OR of 2.66 [95% CI: 1.91; 3.7].

In comparison, the odds of utilization of emergency outpatient services for ACS conditions were 4.89 times [95% CI: 4.1; 5.85] higher among asylum-seeking children than among children of the general population (Table 3). Yet again, 1 to 3 year olds were the most likely age group to make use of emergency services for ACS conditions (OR = 1.19 [95% CI: 1.0; 1.42]).

### Adjusted multiple logistic regression estimates

After controlling for sex, age and period of admission, the estimates for the association of exposure to residence status with both hospitalization for ACS conditions (Table 2) and utilization of emergency outpatient services (Table 3) stayed statistically significantly positive.

In inpatient care, the chance of an asylum-seeking child being admitted for ACS conditions was 1.81 times [1.02; 3.2] higher than that of the control group (Table 2). In emergency outpatient care, the odds of service utilization for ACS conditions by the asylum-seeking population were 4.93 times [4.11; 5.91] the odds of the general population (Table 3). The adjustment for the variables sex, age group and quarterly admission does not have a significant effect on the odds ratios of hospitalization for ACS conditions and utilization of emergency care services for ACS conditions.

## Discussion

Ambulatory care sensitive (ACS) hospitalizations have increasingly been used as an indicator for the availability and quality of ambulatory care on an international level [10]. As a proof of concept, our objective was to assess the differences between asylum-seeking children and children of the general population in a German city with respect to (i) the prevalence of ACS hospitalizations, and (ii) the utilization of emergency outpatient services for ACS conditions. Our main finding is that asylum-seeking minors do have significantly higher odds of hospitalizations for ACS conditions and of utilization of emergency outpatient services for ACS conditions compared to the general population. The observed associations could not be explained by differences in age, sex or different periods of admission throughout the year.

The percentage of asylum-seeking children being hospitalized for ACS conditions was 7% higher than that of the control group, in emergency services use it was nearly 22% higher. This high use of especially emergency outpatient services for ACS conditions among the asylum-seeking population indicates that the preceding entities in ambulatory care were weak, overloaded or insufficient. However, residual confounding by differences in children’s socio-economic status and parents’ health seeking behavior cannot be ruled out (see below).

Nearly 50% of the ACS hospitalizations among asylum-seeking children were attributable to their exposure (*Afe*), but the hospitalizations in this group contributed only to slightly more than 1% to the ACS hospitalizations in the whole population, i.e. the total admissions (*PAF*). While avoiding ACS hospitalizations in the asylum-seeking population is an important goal, addressing ACS hospitalizations in children of the general population would have an even higher public health impact. In outpatient care, nearly 80% of emergency service use for ACS conditions among asylum-seeking children was attributable to their exposure, and about 5% of all emergency service utilizations for ACS conditions could have been averted in absence of the exposure (everything else held constant).

Rates of preventable hospitalization have been used as quality indicators of healthcare provided to populations, as access indicators of primary care and as indicators of outpatient care related to primary care capacity [22]. The main strength of our study was building a useful approach in explicitly measuring deficits in primary care among asylum-seeking populations. The quality indicator ACS hospitalizations allowed quantification of disparities in health services.

The main limitation of our study concerns measurement of influences on ACS hospitalizations outside of the control of the ambulatory care sector, which are often difficult to adjust for. The health of a child can be influenced by many factors beyond the control of a physician, beginning with parental decisions to socioeconomic factors, patient demographics, environment and many more [20]. Many of these factors are difficult to measure and adjust for due to lack of according data. Studies have shown that not only quality of primary care is of importance but also that the characteristics of population and the supply of secondary care resources play a considerable role [1, 23]. Having experienced forced displacement, stress and trauma prior to entering the country of resettlement, forced migrants are more likely to present a different burden of disease and other spectrum of illnesses than the host countries’ population. A particular vulnerability and risk is given regarding specific health conditions, such as infectious, mental and non-communicable chronic diseases [24–26]. Research on the impact of socioeconomic status on hospital use in New York [1] discovered that for all ACS conditions combined, low income areas had rates four times higher, with nearly 70% of the variation explained by area income. Trachtenberg et al. [27] came to a similar conclusion when examining the relationship between respiratory hospitalizations and socioeconomic status and inequities in ambulatory care: the association of socioeconomic status with potentially preventable hospitalizations showed that the odds of being hospitalized in the lowest income group were approximately 3 times higher than the highest income group. This does show that especially socioeconomic status is a variable that does very much have to be taken into account when measuring and using the indicator ACS hospitalizations. Even with adequate primary care, there exists the possibility that a child may be hospitalized for preventable or ACS conditions either because of extraneous factors such as the above mentioned or however an uncontrollable, fulminant exacerbation of their illness [20]. Although ACS conditions have been used in a multitude of analyses to examine avoidability of hospitalizations, they therefore remain an imperfect measure of directly measuring access to primary care [20]. While we had data on children receiving social welfare transfers, it was not possible to disaggregate by residence status in order to determine whether or not these children were asylum seekers. Better identification of migrants in the German health information system is needed [28] to disentangle the impact of migration status separately from that of socioeconomic status.

The role of health seeking behavior and its influence on health care utilization is a point of discussion in this context. As health care systems worldwide differ in many ways, asylum seekers may lack sufficient knowledge and information of and on the structures of our health care system, consequently make more use of emergency outpatient services [4, 29, 30]. Both the different perception and understanding of illnesses and different expectation of treatment could lead to further challenges in the relationship between a medical practitioner and his patient and thus have an influence on the outcome of the treatment. Interviews with health care professionals in 16 European countries showed that expressions of aetiology, symptoms, and pain can make it difficult for an accurate diagnosis to be made, especially when understanding of these concepts greatly differs between the patient and practitioner [4]. This presents the question, if results uncover barriers in access to ambulatory care, fundamental weaknesses in primary care or rather show cultural differences regarding use of health services and medical knowledge. Furthermore, access to appropriate language interpretation is paramount for the appropriate management of asylum seekers’ health [31–33], both in terms of explaining symptoms and in following medical recommendations. In our case, bilingual health care staff, residents of the Patrick Henry Village and lay volunteers provided language interpretation. Shortages of interpreters and communication problems were listed as the largest problem in all administrative meetings of the on-site clinic [7].

Coding behavior in hospitals is a factor that also has to be taken into account. Although the source of data is reliable, the underlying coding behavior in hospitals influences the quality of the data significantly [34]. Overall, however, a largely consistent and standardized coding is to be assumed, due to the German Hospital Reimbursement Act ("*Krankenhausentgeltgesetz*") [34].

Missing identification of primary diagnoses among the outpatient care data could also lead to a distortion of the results, as the diagnosis, which may have ultimately lead to use of emergency care, would trump further ACS conditions within the same hospital case.

In the process of a systematic review on potentially avoidable and ACS hospitalizations among forced migrants [35], only few empirical studies have been found on ACS conditions and hospitalizations among asylum seekers and the broader population group of forced migrants. Preliminary results indicate that the indicator has not been widely used to display differences and disparities in access to ambulatory care among forced migrants and the domestic population.

By conducting this study and demonstrating an association of the indicators ACS hospitalizations and ACS conditions with children’s residence status we aimed to establish a base line for further research. This approach could help to compare the strength of primary care provision in different locations, and allow an objective analysis of regional health system performance from a primary care perspective.

## Conclusion

Our objective was to assess the differences between asylum-seeking children and children of the general population in a German city with respect to the prevalence of ambulatory care sensitive (ACS) hospitalizations and the utilization of emergency outpatient services for ACS conditions. We found that asylum-seeking children had significantly higher odds of hospitalization and of utilization of emergency outpatient services for ACS conditions. The use of emergency outpatient services for ACS conditions among the asylum-seeking population was nearly five times the use of the control group. In inpatient care, the odds for hospitalization of asylum-seeking children in comparison to children of the general population for ACS conditions were significantly higher (around 80%).

Using the concept of ACS conditions allowed measuring the strength of primary care provided to this local asylum-seeking population. We hope to have established a base line for further research, as this approach could help to compare the strength of primary care provision in different locations, quantify observed deficits and allow an objective analysis of regional health system performance from a primary care perspective.

## Abbreviations

ACS: Ambulatory care sensitive
